# Effects of the COVID-19 Pandemic on Gynecological Health: An Integrative Review

**DOI:** 10.1055/s-0042-1742294

**Published:** 2022-02-25

**Authors:** Gisele Vissoci Marquini, Sérgio Brasileiro Martins, Letícia Maria Oliveira, Márcia Maria Dias, Claudia Cristina Takano, Marair Gracio Ferreira Sartori

**Affiliations:** 1 Departament of Gynecology, Escola Paulista de Medicina, Universidade Federal de São Paulo, São Paulo, SP, Brazil

**Keywords:** COVID-19 pandemic, menstrual change, ovarian function, SARS-CoV-2, violence against women, contraception, urogynecology, pandemia de COVID-19, alteração menstrual, função ovariana, SARS-CoV-2, violência contra as mulheres, contracepção, uroginecologia

## Abstract

**Objective** To analyze the existing scientific literature to find out if the coronavirus disease 2019 (COVID-19) pandemic has an effect on gynecological health.

**Search Strategy** We performed an integrative review of articles published between April 2020 and April 2021 on the PubMed, SciELO, and LILACS databases, using *COVID-19* and the following relevant terms: *Menstrual change* ; *Ovarian function* ; *Violence against women* ; *Contraception* ; *HPV* ; *Mental health* ; and *Urogynecology* .

**Selection Criteria** Among the eligible studies found, editorials and primary research articles, which describe the dynamics between severe acute respiratory syndrome coronavirus (SARS-CoV-2) infection (the cause of the COVID-19 pandemic) and gynecological health, were included.

**Data Collection and Analysis** Through qualitative synthesis, data were extracted from the included publications and from guidelines of national and international societies of gynecology.

**Main Results** The 34 publications included in the present study showed that some factors of the SARS-CoV-2 infection, and, consequently, the COVID-19 pandemic, might be associated with menstrual abnormalities, effects on contraception, alterations in steroid hormones, changes in urogynecological care, effects on women's mental health, and negative impact on violence against women.

**Conclusion** The COVID-19 pandemic has significantly impacted the health of women. The scientific community encourages the development of recommendations for specialized care for women and strategies to prevent and respond to violence during and after the COVID-19 pandemic.

## Resumo

**Objetivo** Analisar a literatura científica existente para saber se a pandemia de doença do coronavírus 2019 ( *coronavirus disease 2019* , COVID-19, em inglês) afeta a saúde ginecológica.

**Estratégia de Pesquisa** Realizou-se revisão integrativa de artigos publicados entre abril de 2020 e abril de 2021 nas bases de dados PubMed, SciELO e LILACS, utilizando *COVID-19* e os seguintes termos: *Menstrual change* ; *Ovarian function* ; *Violence against women* ; *Contraception* ; *HPV* ; *Mental health* ; e *Urogynecology* .

**Critério de Seleção** Entre os estudos elegíveis encontrados, foram incluídos editoriais e artigos de pesquisa que descrevem a dinâmica entre a infecção por coronavírus da síndrome respiratória aguda grave ( *severe acute respiratory syndrome coronavirus* , SARS-CoV-2, em inglês), a causa da pandemia de COVID-19, e a saúde ginecológica.

**Coleta e Análise de Dados** Por meio de síntese qualitativa, os dados foram extraídos das publicações incluídas e de diretrizes de sociedades nacionais e internacionais de ginecologia.

**Resultados Principais** As 34 publicações incluídas no estudo mostraram que alguns fatores da infecção por SARS-CoV-2, e, consequentemente, da pandemia de COVID-19, podem estar associados a alterações menstruais, influências na contracepção, alterações em hormônios esteroides, adaptações na assistência uroginecológica, influência na saúde mental da mulher, e impacto negativo na violência contra a mulher.

**Conclusão** A pandemia de COVID-19 teve um impacto significativo na saúde das mulheres. A comunidade científica incentiva o desenvolvimento de recomendações para o atendimento especializado a mulheres, e estratégias para prevenir e combater a violência durante e após a pandemia de COVID-19.

## Introduction

The world is currently in the second year of the coronavuirus disease 2019 (COVID-19) pandemic. Globally, infections by severe acute respiratory syndrome coronavirus (SARS-CoV-2) are continuously rising, with an increasing death toll. Worldwide, the public health response to the pandemic was the imposition of restrictions to minimize the spread of the virus, prevent health system overload, and counter the deficit of personal protective equipment. 1

Although in most cases the symptoms are mild or absent, SARS-CoV-2 infection can lead to serious acute respiratory disease and multiorgan failure. The research community responded to this new disease with a high level of transparency and data sharing, to better understand its origin, pathophysiology, epidemiology and clinical manifestations, with the ultimate goal of developing vaccines, mitigation strategies, as well as potential therapies. 1

Many studies have been performed with different approaches to try to understand the impact of COVID-19 on the materno-fetal binomial. Evidence about COVID-19 and pregnancy has been increasing rapidly since December 2019. However, few studies have raised concerns specifically about gynecology.

The pandemic has significantly impacted gynecological health, causing great mental and physical anguish and huge implications on the overall health of females. 1 2 3 4 5 The aim of the present review is to summarize the current knowledge on the COVID-19 pandemic in gynecology, including what is known about the potential impact of the disease not only on menstrual abnormalities or steroid hormones, but also on reproduction assistance, fertility care, urogynecological assistance, and the dynamics of interpersonal relationships, including, unfortunately, violence against women.

## Methods

The authors performed an integrative review of articles published between April 2020 and April 2021 on the PubMed, SciELO, and LILACS databases, using *COVID-19* and the following relevant terms: *Menstrual change* ; *Ovarian function* ; *Violence against women* ; *Contraception* ; *HPV* ; *Mental health* ; and *Urogynecology* . The present study is a narrative review of the literature available on the coronavirus so far, focusing on gynecological outcomes and strategies, aiming to gather information to safely approach women with suspected or confirmed COVID-19. There were no restrictions regarding language.

Among the eligible studies found, the authors included editorials and primary research articles which describe the dynamics between the SARS-CoV-2 infection (the cause of the COVID-19 pandemic) and gynecological health. Through qualitative synthesis, data were extracted from the included publications and from guidelines of national and international societies of gynecology.

## Results

Table 1 shows relevant outcomes regarding women's health during the COVID-19 pandemic according to the available evidence-based studies, as well as the recommendations made by the authors of the most relevant published studies on COVID-19 and women's disorders.

**Table 1:** Main findings on women's health disorders and issues associated to the COVID-19 pandemic

Author(s)	Women's health subarea	Disorder/issue	Main findings/ recommendations
Godin 3 Sharma et al. 4 Sánchez et al. 5 UN Women 6	Women's mental and physical health	Violence against women	Support care for crisis management/ enlargement of judicial service and health care
UN Women 6	Women's mental health	Family disruptions	Effects on family dynamics/counseling
Phelan et al. 7	Endocrine Gynecology	Menstrual change	Worsening premenstrual symptoms; menstrual volume changes/counseling and treatment availability
Li et al. 8	Women's mental health	Sexual health	Reduced libido/changes in partner relationships/counseling
Mauvais-Jarvis et al. 9 Al-Lami et al. 10 Cattaneo et al. 11 Cagnacci et al. 12 Cagnacci and Xholli 13 Yi et al. 14	Endocrine Gynecology	Menopause	Changes in the immune response and risk of venous thromboembolism/hormonal therapy should be discontinued
Cagnacci et al. 12 Ramírez et al. 15 Fruzzetti et al. 16 Pires et al. 17	Endocrine Gynecology	Contraception	Risk of venous thromboembolism in users of contraception during the COVID-19 pandemic/contraception availability and changes in selected prescriptions if necessary
Gemzell-Danielsson et al. 18 Council of Europe 19 United Nations 20 Brunson 21 Sawhill and Guyot 22 Makins et al. 23 Mantha et al. 24	Endocrine Gynecology	Reproduction	Difficulties in the access to contraception and increase in unintended pregnancies/contraception availability and encouragement of the use of LARCs.
Ciavattini et al. 25 26 ASCCP 27 BSCCP 28 ACOG 29 Arbyn et al. 30	Oncological Gynecology	HPV and cancer prevention	Maintenance or postponement of vaccination, screening programs and treatment for cervical cancer according to the flexibility of each country's restrictions.
Dvash et al. 31	Emergency women's care	Decrease in emergency room arrivals	Strategies to recognize and improve assistance for this secondary impact of the pandemic
Grimes et al. 32 Ferreira et al. 33	Urogynecology	Pelvic floor disorders	Telephysiotherapy and conservative management

Abbreviations: ACOG, American College of Obstetrics and Gynecology; ASCCP, American Society for Colposcopy and Cervical Pathology; BSCCP, British Society for Colposcopy and Cervical Pathology; COVID-19, coronavirus disease 2019; HPV,human papillomavirus; LARCs, long-acting reversible contraceptives; UN Women, United Nations Entity for Gender Equality and the Empowerment of Women.

## Discussion

After the World Health Organization (WHO) characterized the COVID-19 outbreak as a pandemic on March 11, 2020, governments and authorities around the world introduced or intensified restrictive social distancing measures to reduce the spread of the infection. These measures have impacted family dynamics through their effects on income, interpersonal bonds, well-being, and mental health. 6

Violence against women and girls is a human rights violation, and remains a major threat during health emergencies and epidemics. During the COVID-19 pandemic, unfortunately, there has been a remarkable increase in cases of domestic violence against women, with reports from countries such as China, the United Kingdom, the United States, France, Cyprus, Argentina, and Singapore, 3 which has alerted several organizations, researchers, and civil society representatives. 5 6 There have also been reports of sexual exploitation, with landlords physically exploiting female tenants in exchange for cheaper accommodations. The factor attributable to this rise could be the daylong stay at home and the failure to escape an abusive partner, social isolation, the absence of coordination among health, social, and judicial services, and the lack of support care for crisis management. 3 4

Another change that greatly impacts women's health during the pandemic can be observed in their menstrual cycles. It is known that periods of stress and psychological distress, like the current pandemic, can affect women's menstrual cycles. The long-term health implications of this are yet to be determined, and future studies should address it. In September 2020, in Dublin, Ireland, Phelan et al. 7 conducted a study in which 1,031 women of reproductive age were invited to complete an anonymous digital survey via social media. A total of 441 (46%) respondents reported changes in their menstrual cycle, 483 (53%) reported worsening premenstrual symptoms, and 467 (45%) reported reduced libido since the beginning of the pandemic. 7

In a retrospective, cross-sectional study, 2 blood samples from the early follicular phase were tested for sex hormones and anti-Müllerian hormone. Disease severity appears to correlate with greater menstrual changes. Patients with comorbidities such as diabetes, liver disease and malignant tumors, as well as severe cases of confirmed COVID-19 (34% versus 8% of mild or asymptomatic cases) had greater alterations in the menstrual cycle, mainly prolonged cycles or a decrease in volume. 2 However, the average sex hormone concentrations and ovarian reserve did not change significantly in women of child-bearing age with COVID-19. From a biological standpoint, it is plausibe that a suppression in the function of the ovaries causes hormonal changes responsible for menstrual disorders in these patients affected by the severe form of COVID-19. 2

Another study 8 assessed the impact of COVID-19 on partner relationships and sexual and reproductive health in China. From a total of 967 participants, 22% reported a decrease in sexual desire; 41% experienced a decrease in the frequency of sexual intercourse; 30% reported an increase in the frequency of masturbation; and 31% reported a deterioration in partner relationships during the pandemic. Besides that, outpatient services in general gynecology, human reproduction, low-risk prenatal care, family planning, and even access to contraceptives may have been disrupted during the COVID-19 pandemic. 8

According to the data observed so far, it seems that females may show a greater protective response against severe cases of COVID 19 than males. The more favorable immune response may be due to the influence of sex steroids (estrogen, progesterone and androgens) on the patient's immunomodulation and anti-inflammatory response. 9 In addition, the scientific community has already described situations in which the presence of female steroid hormones may provide some advantage over male steroid hormones in the immune response to infectious diseases or aggravations of diseases such as those that are cardiovascular. The same immune response favored by estrogens, progesterone and androgens seems to have occurred in severe cases of COVID-19, which was not maintained in postmenopausal women exposed to COVID-19. 10

If, on the one hand, natural female hormones can hinder the mortality of women by severe COVID-19, on the other hand, exogenous estrogens can increase clotting factors and the risk of thromboembolic events, with a potential consequent increase in mortality, in postmenopausal hormonal therapy users. Hospitalized patients with severe COVID-19 have an activated coagulation demonstrated by high levels of D-dimers (fibrin degradation products), which, in combination with hormonal therapies in postmenopausal women, can favor the development of thromboembolic events. These and other findings even motivated the administration of anticoagulants such as heparin in many protocols for these cases of high levels of D-dimer, in an attempt to reduce mortality, both in men and women. 11 12

In view of these findings, there is a recommendation to suspend hormonal therapies in peri- and postmenopausal women with COVID-19 infection. 12 13 14 15 It is advisable to inform the patient that irregular bleeding may occur with the discontinuation of the hormonal therapy. Shifting from oral to transdermal estrogens (patch, gel, spray) may be considered, but the management of each case should be individualized. 12 When restarting the therapy, due to the lower risk of thromboembolic events, transdermal estrogens should be preferred. 12

The same clinical reasoning can be applied to combined hormonal contraceptives. The use of contraceptive methods that contain only progestogens (oral, intrauterine devices, and implants) is not associated with an increased risk of venous thromboembolism. 12 15 It is recommended that, for users of hormonal therapy or combined oral hormonal contraception who suffer from mild cases of COVID-19 and wish to maintain the use of hormones, is it possible to do so, and, based on the risk factors for venous thromboembolism, and individualizing the management of the cases, the prophylactic use of heparin must be suggested. 15 16 However, low molecular weight heparin should not be used indiscriminately, but under medical supervision, aiming to reduce the risk of venous thromboembolism. 15 16

In addition, in COVID-19 patients with mild symptoms and without severe risk factors, it is also wise to replace combined oral hormonal contraception by contraception containing progestogen alone. Regarding hormonal therapy, when oral, it is recommended to be replaced by transdermal. 16 It is up to gynecologists, like other health professionals, to provide guidance on general measures to prevent the pandemic, including social isolation, use of masks, and hygiene habits such as washing hands with soap and water or using alcohol gel regularly. Gynecologists should also be mindful of recommendations for hormonal therapy and contraception for their patients, whether during or after the pandemic. 17

The pandemic can affect gynecological health not only directly through the SARS-CoV-2 infection, but also through the indirect impact in terms of access to assistance or changes in the dynamics of relationships. Difficulty in accessing health systems during the pandemic has also impacted basic family planning rights. The pandemic may have negatively impacted the basic access to contraceptive care, one of the universal women's health services that minimize gender inequality and grant female autonomy. The secondary impact of the pandemic was observed not only in the difficulty in accessing family planning assistance, but also in effects on the production and transportation of contraceptive products, with the consequent unavailability of state-of-the-art contraceptives, favoring unplanned pregnancies. 1 4 18

In this context, the recommendation of long-acting reversible contraceptives (LARCs) also provides positive social assistance, without the need to go frequently to pharmacies or public centers to get contraceptive, that was evident in the pandemic. By using LARCS, women have less need to access the outpatient family planning system, which, during the pandemic, favored greater health protection and lower economic impact for both the patient and the health system, with possible redistribution of funds to areas related to the fight against COVID-19. 18 19

Therefore, gynecologoical societies, by encouraging access to LARCs, aligned themselves with the pandemic isolation scenario. Unlike short-acting methods such as oral contraceptive pills, LARCS provide effective contraception for years after a single intervention that can mitigate concerns regarding access to and availability of contraceptive services. 18 19 In the context of the COVID-19 pandemic, the therapeutic benefits of LARCs provide an option for treating women with conditions such as heavy menstrual bleeding without an organic cause or dysmenorrhea that minimizes exposure to the hospital environment and reduces lengthy waits for surgical appointments. 18 19

In the field of Oncological Gynecology, the scientific community recognized the high level of concern with outpatient care during the COVID-19 pandemic. Cancer prevention and early diagnosis are basic pillars of gynecological care; unfortunately, they had to be postponed or suspended during the peak of the COVID-19 health crisis due to changes in the structures of medical institutions. In relation to cervical cancer, which fortunately presents an evolution that is not very rapid evolution, several societies supported the momentary postponement of follow-ups at the height of the pandemic, without affecting the safety of the disease's evolution. These recommendations should to be applied at the discretion of the services, with respect to the regionality and individuality of each case. But overall, societies such as the European Federation for Colposcopy (EFC) and the European Society of Gynecological Oncology (ESGO), have advised that human papillomavirus (HPV) vaccination, screening, colposcopy and outpatient lower genital tract surgery programs, as well as follow-up, should be rescheduled to a safer moment of the health crisis, without effectively compromising the safety of these measures. 25 [Bibr JR210239-26] [Bibr OR210239-27] [Bibr OR210239-28] [Bibr OR210239-29] - 30 Cervical cancer screening and HPV vaccination may continue in countries with no cases or sporadic cases of COVID-19, and should be delayed in countries with clusters of cases and/or community transmission of COVID-19 to minimize the progression of the pandemic. 25

In addition, according to the EFC and ESGO, in case of suspension of elective diagnostic and therapeutic procedures, the timing of the management of abnormal histopathological results in cervical biopsies is dictated by the risk of progression of preinvasive lesions. Those patients may need a subsequent diagnostic procedure or outpatient surgical treatment (such as cervical, vaginal, or vulvar excision) or a complete assessment to plan the staging and treatment for invasive cancer. 25 Patients with a histopathologic diagnosis of invasive disease from cervical, vaginal, or vulvar biopsy should be contacted within two weeks. Additionally, patients with symptoms suggestive of lower genital tract cancers should also be evaluated within two weeks. A histopathological diagnosis of a low-grade intraepithelial lesion from a cervical, vaginal, or vulvar biopsy/excision enables physicians to postpone the contact up to 12 months. 25

Another relevant consequence of the pandemic was the decrease in the search for assistance in gynecology emergency rooms. The reduced flow of patients seeking urgent care in gynecology may have screened cases that are really relevant for care, with real need, but, on the other hand, it may have decreased assistance in really urgent cases such as hemorrhagic, infectious acute abdomen, pelvic inflammatory disease, among other situations of emergency care. 31 32 An example of this contingency of emergency care in gynecology was evidenced in a retrospective cohort study that assessed the impact of the COVID-19 pandemic on the diagnosis, treatment, and complications of women who presented with tubal ectopic pregnancy. 31 The authors found a higher rate of ruptured ectopic pregnancy, with ultrasonographic evaluation of more cases of high volume of fluid in the abdomen during the COVID-19 pandemic than before (53% versus 17%; *p* = 0.01). 31 Evidence may have pointed to this change in behavior in seeking assistance in gynecology emergency rooms, but data are still very limited.

Likewise, the management of female pelvic floor disorders such as pelvic organ prolapse and urinary incontinence was a challenge due to the COVID-19 pandemic. Multidisciplinary care in urogynecology with pelvic physiotherapy in outpatient or surgical care had to be readapted and urogynecologists faced challenges to safely continue their work, considering the adoption of social distancing measures during the pandemic. Some guidelines have already been published by urogynecology associations; however, the recommendations should be applied at the discretion of the governments of each country, and according to each moment of the health crisis and each individual case. 32 33 General measures, behavioral therapies, medical and physiotherapy guidelines added to the conservative treatment were valuable as first-line treatments, often virtual. Certain situations required different treatments in the virtual environment, while others required a personal visit, despite the risks of COVID-19 transmission. Even after the climax of the health crisis, with the possibility of returning to surgical treatments, the multidisciplinary conservative approach is recommended as the first line of treatment to continue care in this area even during and after the COVID-19 pandemic, with the perspective of improving the quality of life of these patients. 32 33

In summary, women's health and the COVID-19 pandemic are extremely linked; however, the impact of the pandemic on assistance and public health strategies is not yet clear. The strength of the study is a review focused on gynecology. Due to the nobility of the materno-fetal binomial, most of the articles published on an urgent basis during the pandemic were directed to maternofetal care. The gynecological patient deserves attention with a collection of studies specifically aimed at health care, not only Obstetrics but also in Gynecology, for non-pregnant women associated with the pandemic.

A limitation of the study is the non-systematic methodology. The authors believe that the limitation of the non-systematized methodology does not detract from the study because the objective was to shed light on women's health care during and after the pandemic. Unfortunately, due to the scarcity of randomized studies on COVID-19 and women's health, far from establishing guidelines, the authors only suggest a possible way of assisting women during and after this moment of health challenge. Besides that, the academic articles report the need to think about strategies during and after the pandemic, given the probability that the vulnerable conditions of women will continue, mainly related to violence. As the WHO Director General has stated in his COVID-19 press briefings, “No one will be safe unless everyone is safe”. 34

## Conclusion

This virus has reminded us that we are all inexorably linked in our common humanity. So far, in a way that is not yet fully clear, the COVID-19 pandemic affects gynecological health. The provision of effective women's health care strategies around the world will result in a healthier future for all. In addition, humanization and empathy in gynecological care, as in any other field, are always welcome, especially in times like those of a pandemic. Gynecological measures and guidelines are encouraged to safeguard the sexual and reproductive health of young people during this pandemic. Moving forward, it seems reasonable to hope that, with further developments and ongoing initiatives, access to LARCs will become a possibility for women across the globe even during the pandemic and after it. The authors suggest continuous strong reforms, policies, and measures such as telemedicine, hotlines, and online counseling forums, to counter the social disturbances related to women's health. Finally, based on the multiple tasks of women in society, the authors wish to rephrase the wise statement made by the WHO Director General as “No one will be safe unless every *woman* is safe”.

## References

[1] Madjunkov M Dviri M Librach C A comprehensive review of the impact of COVID-19 on human reproductive biology, assisted reproduction care and pregnancy: a Canadian perspective J Ovarian Res 2020 13 01 140 10.1186/s13048-020-00737-1 33246480 PMC7694590

[2] Li K Chen G Hou H Analysis of sex hormones and menstruation in COVID-19 women of child-bearing age Reprod Biomed Online 2021 42 01 260 267 10.1016/j.rbmo.2020.09.020 33288478 PMC7522626

[3] Godin M As cities around the world go on lockdown, victims of domestic violence look for a way out Time. 2020Mar 18 [cited 2021 Apr 23]. Available from:https://time.com/5803887/coronavirus-domestic-violence-victims

[4] Sharma P Sharma S Singh N COVID-19: Endangering women's mental and reproductive health Indian J Public Health 2020 64(Supplement): cited2021Apr23 [Internet]S251 S252 https://www.ijph.in/temp/IndianJPublicHealth646251-4759005_131310.pdf 32496270 10.4103/ijph.IJPH_498_20

[5] Sánchez O R Vale D B Rodrigues L Surita F G Violence against women during the COVID-19 pandemic: An integrative review Int J Gynaecol Obstet 2020 151 02 180 187 10.1002/ijgo.13365 32880941 PMC9087782

[6] UN Women. Prevention: violence against women and girls & COVID-19 [Internet] 2020[cited 2021 Apr 17]. Available from:https://www.unwomen.org/-/media/headquarters/attachments/sections/library/publications/2020/brief-prevention-violence-against-women-and-girls-and-covid-19-en.pdf

[7] Phelan N Behan L A Owens L The impact of the COVID-19 pandemic on women's reproductive health Front Endocrinol (Lausanne) 2021 12 642755 10.3389/fendo.2021.642755 33841334 PMC8030584

[8] Li G Tang D Song B Impact of the COVID-19 pandemic on partner relationships and sexual and reproductive health: cross-sectional, online survey study J Med Internet Res 2020 22 08 e20961 10.2196/20961 32716895 PMC7419154

[9] Mauvais-Jarvis F Klein S L Levin E R Estradiol, progesterone, immunomodulation, and COVID-19 outcomes Endocrinology 2020 161 09 127 10.1210/endocr/bqaa127 PMC7438701 32730568

[10] Al-Lami R A Urban R J Volpi E Algburi A MA Baillargeon J Sex Hormones and Novel Corona Virus Infectious Disease (COVID-19) Mayo Clin Proc 2020 95 08 1710 1714 10.1016/j.mayocp.2020.05.013 32753145 PMC7256539

[11] Cattaneo M Bertinato E M Birocchi S Pulmonary embolism or pulmonary thrombosis in COVID-19? Is the recommendation to use high-dose heparin for thromboprophylaxis justified? Thromb Haemost 2020 120 08 1230 1232 10.1055/s-0040-1712097 32349132 PMC7516356

[12] board of the Italian Menopause Society Cagnacci A Bonaccorsi G Gambacciani M Reflections and recommendations on the COVID-19 pandemic: Should hormone therapy be discontinued? Maturitas 2020 138 76 77 10.1016/j.maturitas.2020.05.022 32565009 PMC7301099

[13] Cagnacci A Xholli A Age-related difference in the rate of coronavirus disease 2019 mortality in women versus men Am J Obstet Gynecol 2020 223 03 453 454 10.1016/j.ajog.2020.05.039 32460973 PMC7246049

[14] Yi Y Lagniton P NP Ye S Li E Xu R H COVID-19: what has been learned and to be learned about the novel coronavirus disease Int J Biol Sci 2020 16 10 1753 1766 10.7150/ijbs.45134 32226295 PMC7098028

[15] Ramírez I De la Viuda E Baquedano L Managing thromboembolic risk with menopausal hormone therapy and hormonal contraception in the COVID-19 pandemic: Recommendations from the Spanish Menopause Society, Sociedad Española de Ginecología y Obstetricia and Sociedad Española de Trombosis y Hemostasia Maturitas 2020 137 57 62 10.1016/j.maturitas.2020.04.019 32498938 PMC7200366

[16] Fruzzetti F Cagnacci A Primiero F Contraception during Coronavirus-Covid 19 pandemia. Recommendations of the Board of the Italian Society of Contraception Eur J Contracept Reprod Health Care 2020 25 03 231 232 10.1080/13625187.2020.1766016 32436739

[17] Pires A LR Batista J G Aldrighi J M Risk of venous thromboembolism in users of contraception and menopausal hormone therapy during the COVID-19 pandemic Rev Assoc Med Bras (1992) 2020 2020(66, Suppl 02)22 26 10.1590/1806-9282.66.S2.22 32965350

[18] Gemzell-Danielsson K Kubba A Caetano C Faustmann T Lukkari-Lax E Heikinheimo O More than just contraception: the impact of the levonorgestrel-releasing intrauterine system on public health over 30 years BMJ Sex Reprod Health 2021 47 03 228 230 10.1136/bmjsrh-2020-200962 PMC8292574 33514606

[19] Council of Europe Parliamentary Assembly. Resolution 2331: empowering women: promoting access to contraception in Europe [Internet] 2020[cited 2021 May 01]. Available from:http://www.europeanrights.eu/public/atti/Resolution_2331_(2020)_ENG.pdf

[20] United Nations Sustainable Development Goals [Internet] 2021[cited 2021 May 01]. Available from:https://www.un.org/sustainabledevelopment/sustainable-development-goals/

[21] Brunson J Tool of economic development, metric of global health: Promoting planned families and economized life in Nepal Soc Sci Med 2020 254 112298 10.1016/j.socscimed.2019.05.003 31113652

[22] Sawhill I V Guyot K Preventing unplanned pregnancy: lessons from the States [Internet] Washington (DC) Brookings Institution 2019[cited 2021 May 01]. Available from:https://www.brookings.edu/wp-content/uploads/2019/06/Preventing-Unplanned-Pregnancy-2.pdf

[23] FIGO Contraception and Family Planning Committee Makins A Arulkumaran S Contraception F IGO The negative impact of COVID-19 on contraception and sexual and reproductive health: Could immediate postpartum LARCs be the solution? Int J Gynaecol Obstet 2020 150 02 141 143 10.1002/ijgo.13237 32449192 PMC9087606

[24] Mantha S Karp R Raghavan V Terrin N Bauer K A Zwicker J I Assessing the risk of venous thromboembolic events in women taking progestin-only contraception: a meta-analysis BMJ 2012 345 e4944 10.1136/bmj.e4944 22872710 PMC3413580

[25] Ciavattini A Delli Carpini G Giannella L European Federation for Colposcopy (EFC) and European Society of Gynaecological Oncology (ESGO) joint considerations about human papillomavirus (HPV) vaccination, screening programs, colposcopy, and surgery during and after the COVID-19 pandemic Int J Gynecol Cancer 2020 30 08 1097 1100 10.1136/ijgc-2020-001617 32487685 PMC7418593

[JR210239-26] Ciavattini A Delli Carpini G Giannella L Expert consensus from the Italian Society for Colposcopy and Cervico-Vaginal Pathology (SICPCV) for colposcopy and outpatient surgery of the lower genital tract during the COVID-19 pandemic Int J Gynaecol Obstet 2020 149 03 269 272 10.1002/ijgo.13158 32270477 PMC9087780

[OR210239-27] ASCCP interim guidance for timing of diagnostic and treatment procedures for patients with abnormal cervical screening tests [Internet] 2020[cited 2021 May 01]. Available from:https://www.asccp.org/covid-19

[OR210239-28] British Society for Colposcopy and Cervical Pathology COVID-19 Pandemic - BSCCP Colposcopy Guidance [Internet] 2020[cited 2021 May 01]. Available from:https://www.bsccp.org.uk/assets/file/uploads/resources/Colposcopy_guidance__COVID_19_pandemic.V3.pdf

[OR210239-29] American College of Obstetricians and Gynecologists COVID-19 FAQs for obstetrician–gynecologists, gynecology [Internet] Washington (DC) ACOG 2020[cited 2021 May 01]. Available from:https://www.acog.org/clinical-information/physician-faqs/covid19-faqs-for-ob-gyns-gynecology

[30] Arbyn M Bruni L Kelly D Tackling cervical cancer in Europe amidst the COVID-19 pandemic Lancet Public Health 2020 5 08 e425 10.1016/S2468-2667(20)30122-5 32673570 PMC7357980

[31] Dvash S Cuckle H Smorgick N Vaknin Z Padoa A Maymon R Increase rate of ruptured tubal ectopic pregnancy during the COVID-19 pandemic Eur J Obstet Gynecol Reprod Biol 2021 259 95 99 10.1016/j.ejogrb.2021.01.054 33636621 PMC7968738

[32] Grimes C L Balk E M Crisp C C A guide for urogynecologic patient care utilizing telemedicine during the COVID-19 pandemic: review of existing evidence Int Urogynecol J Pelvic Floor Dysfunct 2020 31 06 1063 1089 10.1007/s00192-020-04314-4 PMC7185267 32342112

[33] Ferreira C HJ Driusso P Haddad J M A guide to physiotherapy in urogynecology for patient care during the COVID-19 pandemic Int Urogynecol J Pelvic Floor Dysfunct 2021 32 01 203 210 10.1007/s00192-020-04542-8 PMC7521075 32986147

[34] Director General Press Briefings COVID-19 [press release] 2020[cited 2021 May 01]. Available from:https://www.who.int/emergencies/diseases/novel-coronavirus-2019/media-resources/press-briefings

